# *Mycobacterium marinum* MgtC Plays a Role in Phagocytosis but is Dispensable for Intracellular Multiplication

**DOI:** 10.1371/journal.pone.0116052

**Published:** 2014-12-29

**Authors:** Claudine Belon, Laïla Gannoun-Zaki, Georges Lutfalla, Laurent Kremer, Anne-Béatrice Blanc-Potard

**Affiliations:** 1 Laboratoire de Dynamique des Interactions Membranaires Normales et Pathologiques, Universités Montpellier 2 et 1, Place Eugène Bataillon, 34095, Montpellier, Cedex 05, France; 2 Centre National de la Recherche Scientifique, UMR5235, Montpellier, France; 3 Institut national de la santé et de la recherche médicale, Montpellier, France; Centre National de la Recherche Scientifique - Université de Toulouse, France

## Abstract

MgtC is a virulence factor involved in intramacrophage growth that has been reported in several intracellular pathogens, including *Mycobacterium tuberculosis* and *Salmonella enterica* serovar Typhimurium. MgtC participates also in adaptation to Mg^2+^ deprivation. Herein, we have constructed a *mgtC* mutant in *Mycobacterium marinum* to further investigate the role of MgtC in mycobacteria. We show that the *M. marinum mgtC* gene (*Mma mgtC*) is strongly induced upon Mg^2+^ deprivation and is required for optimal growth in Mg^2+^-deprived medium. The behaviour of the *Mma mgtC* mutant has been investigated in the *Danio rerio* infection model using a transgenic reporter zebrafish line that specifically labels neutrophils. Although the *mgtC* mutant is not attenuated in the zebrafish embryo model based on survival curves, our results indicate that phagocytosis by neutrophils is enhanced with the *mgtC* mutant compared to the wild-type strain following subcutaneous injection. Increased phagocytosis of the mutant strain is also observed *ex vivo* with the murine J774 macrophage cell line. On the other hand, no difference was found between the *mgtC* mutant and the wild-type strain in bacterial adhesion to macrophages and in the internalization into epithelial cells. Unlike the role reported for MgtC in other intracellular pathogens, *Mma* MgtC does not contribute significantly to intramacrophage replication. Taken together, these results indicate an unanticipated function of *Mma* MgtC at early step of infection within phagocytic cells. Hence, our results indicate that although the MgtC function is conserved among pathogens regarding adaptation to Mg^2+^ deprivation, its role towards phagocytic cells can differ, possibly in relation with the specific pathogen's lifestyles.

## Introduction

MgtC is a virulence factor common to several intracellular pathogens [1]. It was first described in *Salmonella enterica* serovar Typhimurium (*S.* Typhimurium) as required for intramacrophage multiplication and systemic infection in mice [2]–[4]. Later, it was described as a critical factor for the intramacrophage growth of *Mycobacterium tuberculosis*, *Brucella suis*, *Yersinia pestis*, *Burkholderia cenocepacia* and *Salmonella enterica* serovar Typhi [5]–[9]. Despite its importance in the virulence of various bacterial pathogens, the mechanism by which MgtC promotes intracellular growth remains unknown. *Salmonella* MgtC has been recently shown to directly interact with bacterial F_1_F_o_ ATP synthase, thereby altering its ability to translocate protons and to couple translocation to ATP synthesis [10]. Hence, modulation of F-ATP synthase activity is proposed to drive the ability of MgtC to promote intramacrophage replication.

MgtC has been associated to adaptation to low Mg^2+^ environments in broth media in the various pathogens mentioned above, based on the observation that growth of the corresponding *mgtC* mutants is impaired in Mg^2+^ deprived media [1]. Moreover, expression of *mgtC* gene is highly induced by deprivation of external Mg^2+^ concentration in *S.* Typhimurium and *Y. pestis* [11], [12], which are two organisms where *mgtC* is cotranscribed with the Mg^2+^ transporter encoded by *mgtB*. However, *mgtC* has been shown to be only slightly induced by Mg^2+^ limitation in *M. tuberculosis* [13], [14], suggesting that Mg^2+^ may regulate MgtC only in bacterial species where it is cotranscribed with a Mg^2+^ transporter.

MgtC is a membrane-associated protein that harbors a conserved hydrophobic N-terminal and a more divergent soluble C-terminal domain that exhibits conservation among proteins from intracellular pathogens as *S.* Typhimurium and *M. tuberculosis* [15]. MgtC has been shown to play a role in *M. tuberculosis* virulence in macrophage and mice infection models [5] but the contribution of *mgtC* in mycobacterial physiology and virulence needs further investigation. Because MgtC is conserved between *M. tuberculosis* and *Mycobacterium marinum*, we aimed to address its role in *M. marinum* virulence, as well as its regulation by magnesium. *M. marinum* is closely related to *M. tuberculosis* not only in its pathology but also genetically [16], [17], and has been increasingly used as a suitable model for understanding the pathogenesis of tuberculosis. As a natural host for this pathogen, zebrafish provides a powerful vertebrate model to study *M. marinum* pathogenesis. Moreover, because of their genetic tractability and optical transparency, zebrafish embryos have been successfully used to investigate host-bacteria interactions and the role of innate immunity during *M. marinum* infection [18]–[20].

The present results indicate that *Mma mgtC* transcription is strongly induced by magnesium deprivation and, unexpectedly, that *Mma* MgtC appears dispensable for intramacrophage replication but plays a role in phagocytosis, a phenotype first uncovered in zebrafish embryos.

## Materials and Methods

### Bacterial strains and growth culture conditions

*Mycobacterium marinum* M and *Mycobacterium smegmatis* mc^2^155 strains were grown at 30°C and 37°C, respectively, in Sauton's medium containing 0.025% of tyloxapol (Sigma) or on Middlebrook 7H10 agar plates supplemented with 10% Oleic acid-Albumin-Dextrose-Catalase (OADC) enrichment, in the presence of kanamycin (25 µg/ml), hygromycin (80 µg/ml) and zeocin (25 µg/ml), when required. Low magnesium medium was obtained by replacing the magnesium sulfate in the Sauton's medium by a similar concentration of potassium sulfate. *Escherichia coli* (DH5α) was used for cloning and was grown in LB medium with zeocin (25 µg/ml) at 37°C.

### RNA extraction and qRT-PCR

RNA was prepared from 5 ml of mid-logarithmic bacterial cultures (grown in Sauton's medium containing or not magnesium). Bacteria were harvested, resuspended in 1 ml of RNA protect reagent (Qiagen) and incubated for 1 h at room temperature. Bacteria were centrifuged and resuspended in 1 ml of RLT buffer from RNeasy Mini kit (Qiagen), transferred in a Lysing matrix B tube (MP Bio) and disrupted with a bead-beater apparatus (3 times, 45 sec, maximal speed). RNA was purified with the RNeasy kit, according to manufacturer's instructions. DNA was further removed using DNAseI (Invitrogen). cDNA was produced using Superscript III reverse transcriptase (Invitrogen). Controls without reverse transcriptase were done on each RNA sample to rule out DNA contamination. Quantitative real-time PCR was performed using an in-house SYBR Green mix and a 480 light cycler instrument (Roche). PCR conditions were as follows: 3 min denaturation at 98°C, 45 cycles of 98°C for 5 sec, 68°C for 10 sec and 72°C for 10 sec. The *sigA* gene (*MMAR_2011*) was used as internal control. The sequences of primers used for qRT-PCR are listed in S1 Table.

### Construction of *M. marinum mgtC* mutant and complemented strains

A strain deleted for the *mgtC* gene was constructed in *M. marinum* M using mycobacteriophage-mediated allelic exchange to replace *mgtC* by a hygromycin cassette. The construction of the allelic exchange substrate (AES) phasmid, the preparation of phage and the phage transduction was performed as described [21]. One kb long sequences upstream and downstream of *mgtC* were cloned into the pJSC347 on both sides flanking the hygromycin cassette. The mutant was checked by PCR and southern blot using probes corresponding to the *mgtC* and hygromycin genes. Complementation of the *mgtC* mutant was performed using a chromosomal copy of the *mgtC* gene placed under the control of its own promoter. A DNA fragment including the *mgtC* gene and 840 bp upstream (which contains the intergenic region between *MMAR_2685* and *MMAR_2686* as well as *MMAR_2687* gene) was amplified by PCR using primers pMV306-*Xba*I-5′ and pMV306-*Hind*III-3′ and was cloned at the *Xba*I and *Hind*III sites of the integrative vector pMV306 [22]. The resulting plasmid was electroporated in the *mgtC* mutant to integrate the *mgtC* gene with its upstream sequences at the chromosomal *attB* site with selection on 7H10 agar plates containing kanamycin. As a control, the empty pMV306 vector was also electroporated into the wild-type strain and the *mgtC* mutant strain.

### Zebrafish embryos injections and microscopy

Experiments were performed using the *golden* zebrafish mutant [23] or the *Tg(mpx:EGFP)* where GFP is specifically expressed in neutrophils [24]. Zebrafish were raised and maintained according to standard procedures [25]. Embryos were obtained from pairs of adult fish by natural spawning and raised at 28.5°C in tank water. Ages are expressed as hours or days post fertilization (hpf or dpf). Mycobacteria from mid-log phase cultures grown in Sauton's medium were washed twice in PBS, resuspended in PBS and homogenized with a 26G syringe (five times) at an OD_600_ of 3. Zebrafish larvae of 30 hpf (caudal vein injection) or 3 dpf (subcutaneous injection) were anesthetized by immersion in buffered tricaine (Sigma A-5040) and manually dechorionated, if needed. For subcutaneous infection, embryos were fed with tetrahymenas from 5 dpf to the end of the experiment, 3 times per week. They were injected with 1 nl of bacterial suspension using a Tritech Research digital microINJECTOR (MINJ-D). To determine bacterial loads in infected embryos, groups of two infected embryos were collected and dissociated using the BD mycoprep Kit. Suspensions were homogenized with a 26G syringe (five times) and dilutions were plated on Middlebrook 7H10 supplemented with 25 µg/ml of kanamycin to determine the colony forming units (CFU). All quantitative results are from triplicate experiments. Wide-field, bright-field and fluorescence live microscopy of infected embryos were performed using an Olympus MVX10 epifluorescence microscope. Images were acquired with a digital color camera (Olympus XC50) and processed using CellSens (Olympus). Fluorescence filters cubes (FITC-MVX10 and TRITC-MVX10) were used to detect green and red light, respectively. Confocal fluorescence microscopy was performed using a Leica DM2500CSQ upright microscope with a Leica TCS SPE confocal scan head, differential interference contrast (DIC) optics and a SuperZGalvo SPE z-step controller. For fixed-sample observations, embryos in 50% glycerol in PBS were mounted flat onto depression transparent slides with a coverslip and observed with a 10× Leica Apo 0.3 NA, 40× Leica Apo oil 1.15 NA or 63× Leica Apo oil 1.33 NA objectives. Overlays of fluorescent and DIC images and 2D reconstructions of image stacks were processed and assembled using LAS-AF software. Final image analysis and visualization were performed using GIMP 2.8 freeware to adjust levels and brightness and to remove out-of-focus background fluorescence.

### Ethics statement

All animal experiments described in the present study were conducted at the University Montpellier 2 according to European Union guidelines for handling of laboratory animals (http://ec.europa.eu/environment/chemicals/lab_animals/home_en.htm) and were approved by the Direction Sanitaire et Vétérinaire de l'Hérault and Comité d'Ethique pour l'Expérimentation Animale under reference CEEA-LR-13007. The breeding of adult fish adhered to the international guidelines specified by the EU Animal Protection Directive 2010/63/EU and adult zebrafish were not sacrificed for this study. For survival curves, cardiac rhythm was used as a clinical criteria to fix the endpoint at which embryos are euthanized using the anaesthetic Tricaine up to a lethal dose. Condition of the infected animals was monitored three times a day. For CFU counts, embryos were anaesthetized with Tricaine up to a lethal dose before lysis with triton. Embryos that survive infection were anaesthetized with Tricaine up to a lethal dose before bleach treatment. For live imaging analysis, embryos were anaesthetized with Tricaine. For microscopic observation of fixed samples, embryos were fixed with 4% paraformaldehyde after being anaesthetized with Tricaine up to a lethal dose.

### Macrophage infection assays

J774 cells were maintained at 37°C in 5% CO_2_ in Dulbecco's modified Eagle medium (DMEM) (Gibco) supplemented with 10% fetal bovine serum (FBS) (Gibco). J774 cells were allowed to adhere in a 24-well plate at a density of 5×10^4^ cells/well for 24 h at 37°C in 5% CO_2_.

For infection, *M. marinum* cultures grown exponentially in Sauton's medium (OD_600_ around 0.8 to 1) were centrifuged, washed in phosphate buffer saline (PBS) and bacterial clumps were disrupted by 8 successive passages through a 26G needle. The remaining aggregates were then eliminated with a short spin (1 min at 1,100 rpm). For experiments including the *wbbl2* mutant as control, mycobacteria were grown on Sauton's agar plates (to induce LOS production in the wild-type strain) containing 10% OADC enrichment for 5–7 days as described [26]. Bacterial lawns were scraped off the plates, resuspended in 1 ml of PBS and clumps were disrupted as indicated above followed by low speed centrifugation to eliminate aggregates. In all experiments, macrophages were infected at a multiplicity of infection (MOI) of approximately 2. The infection was allowed to proceed for 3 h at 32°C in 5% CO_2_ prior to exposure to 200 µg/ml gentamicin for 60 min to kill the remaining extracellular bacteria. Infected cells were then washed three times with PBS. Cells were then lysed with 0.1 ml of 1% Triton X-100 in PBS and the number of intracellular mycobacteria counted by plating appropriate dilutions onto Middlebrook 7H10 agar plates. To evaluate the numbers of phagocytosed bacteria, the ratio between the count of internalized bacteria and the count of bacteria in the inoculum was determined. For kinetic experiments, earlier infection times (0.5, 1, and 2 h) were also assessed. To evaluate the multiplication rate, after the three washes with PBS, infected cells were incubated for 5 days in DMEM medium supplemented with 20 µg/ml gentamicin (a concentration that prevents growth of *M. marinum* strains) at 32°C in 5% CO_2_ prior to cell lysis and bacterial enumeration. The ratio between the number of internalized bacteria at day 5 and the number of internalized bacteria after 3 h infection was calculated. To evaluate bacterial adherence, the same procedure was applied, except that J774 cells were incubated 30 min with 10 µg/ml cytochalasin D prior to infection or were incubated at 4°C during the phagocytic time. All results are derived from a least three independent experiments where strains are tested in triplicate.

To visualize and count the number of bacterium-containing macrophages, a similar protocol was used except that macrophages were allowed to adhere on coverslips prior to infection with fluorescent bacteria carrying the pMV261_mCherry (Table 1). Following exposure to gentamycin, infected cells were washed three times in PBS and labeled with fluorescein-conjugated phalloidin (Sigma), according to the manufacturer's instructions to visualize the shape of the cells. Stained cells were then observed using a Zeiss Axioimager and quantification of mycobacterium-residing cells was determined by counting 20 fields per coverslip. Each strain was analyzed in triplicate.

**Table 1:** Bacterial strains and plasmids.

Strains, plasmids, phages	Description or phenotype	Source/Ref.
***Mycobacterium marinum*** ** strains**		
Mma M	wild-type	[16]
Mma Δ*mgtC*	Δ*mgtC*	This study
Mma Δ*mgtC*+*mgtC*	Δ*mgtC*+*attB*::*mgtC*	This study
Mma Δ*wbbl2*	Δ*wbbl2*	[26]
***Mycobacterium smegmatis*** ** strains**		
mc^2^155	Electroporation-proficient *ept* mutant of *M. smegmatis* strain mc^2^6	[49]
**Plasmid**		
pJSC347	Vector for cloning allelic-exchange substrates to be used for specialized transduction, Hyg^R^	[50]
pMV306	Single-copy-integrating vector, Kan^R^	[22]
pMV306-mgtC	pMV306 carrying the *mgtC* gene and 840 bp upstream, Kan^R^	This study
pMV261-mCherry	pMV261 carrying mCherry under the control of the *hsp60* promoter, Zeo^R^	[51]
**Phage**		
phAE159	Conditionally replicating shuttle phasmid derived from the lytic mycobacteriophage TM4	Kind gift from W.R. Jacobs

### HeLa cells infection assays

HeLa cells were maintained at 37°C in 5% CO_2_ in DMEM supplemented with 10% fetal bovine serum. HeLa cells were allowed to adhere in 24-well plates at a density of 5×10^4^ cells per well for 24 h at 37°C in 5% CO_2_. For infection, mycobacteria were prepared as described above from Sauton's agar plates because the *wbbl2* mutant was also included in the experiment. The internalization protocol was similar to the phagocytosis protocol.

### Extraction and analysis of mycobacterial apolar lipids

*M. marinum* strains were grown to mid-log growth phase in Sauton's medium containing or not magnesium sulfate and labeled with 0.5 µCi/ml [1-^14^C]propionate (specific activity of 54 mCi/mmol, American Radiolabeled Chemicals, Inc) for 24 h. The non-polar fraction was separated from the polar fraction as described [27]. The [1-^14^C]-propionate-labeled apolar lipids were extracted by adding 2 ml of CH_3_OH/0.3% NaCl (100/10; v/v) and 2 ml of petroleum ether to the cell pellet followed by stirring for 1 h. After centrifugation, the upper petroleum ether layer was removed and 2 ml of petroleum ether was added to the lower phase. The combined petroleum ether extracts were then evaporated under a nitrogen stream to yield apolar lipids that were resuspended in CH_2_Cl_2_ and analyzed by TLC using silica gel plates (5735 silica gel 60F254; Merck, Darmstadt, Germany). Equivalent amounts of lipids (20 000 cpm) were spotted on TLC plates that were run in various solvent systems: Petroleum ether/diethylether 9∶1 (v/v) for PDIM; chloroform/methanol 19∶1 (v/v) for TDM and PGL and chloroform/methanol 99∶1 (v/v) for PAT. TLCs were exposed to a Kodak Biomax MR film for 24 h.

### Statistical analyses

For zebrafish embryos infections, statistical analyses were performed using GraphPad Prism 4 (Graphpad Software). Survival curves were compared between two groups by using the logrank test. For cells infection assays, averages were compared by using an unpaired Student *t*-test. Only *P*-values <0.05 were considered as statistically significant.

## Results

### Identification of a MgtC-like protein in *M. marinum* and construction of *Mma mgtC* mutant strain

**Figure 1:**
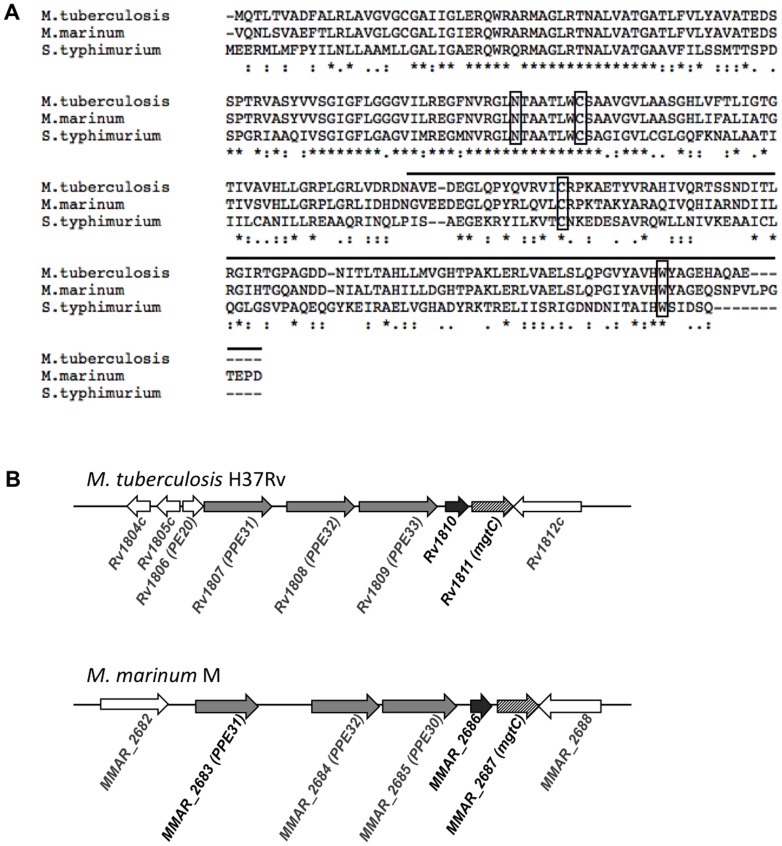
Alignment of mycobacterial MgtC proteins and genetic environment of *mgtC* gene. (A) Alignment of *S.* Typhimurium MgtC (Accession Number AAL22622.1), *M. tuberculosis* MgtC (Accession Number NP_216327.1) and *M. marinum* MgtC (Accession Number ACC41130.1) using ClustalW. The upper line indicates the soluble C terminal part. Rectangles indicate four conserved residues that have been shown to be essential for *Salmonella* MgtC function. (B) Genetic environment of *mgtC* gene (striped arrows) in *M. tuberculosis* and *M. marinum* genomes. In both species, the *mgtC* gene is adjacent to *Rv1810* that is homologous to *MMAR_2686* (black arrows) and to PPE genes (grey arrows). The *MMAR_2688* gene is homologous to *Rv1812c*.

An MgtC-like protein is encoded by the *MMAR_2687* gene in *M. marinum* genome (http://mycobrowser.epfl.ch/marinolist.html). The *Mma* MgtC protein shares strong similarity to the *Mtb* MgtC protein (82% identity, 90% similarity) (Fig. 1A), the major difference residing in the very C-terminal end of the protein. The sequence of *Mma* MgtC is also well conserved with that of *S.* Typhimurium MgtC in the N-terminal half of the protein (61% identity, 73% similarity), but more divergent in the C-terminal half (102 last a.a. 20% identity, 37% similarity). In addition, *Mma* MgtC harbors four conserved residues that have been shown to be important for *Salmonella* MgtC function (Fig. 1A) [28]. The genomic organization around *mgtC* is conserved between *M. marinum* and *M. tuberculosis* (Fig. 1B). In both species, *mgtC* is located downstream of several genes coding for PPE proteins and is adjacent to a gene coding for a protein of unknown function (Rv1810 or MMAR_2686). This genomic organization differs from the one of *S.* Typhimurium where *mgtC* is in operon with a gene that encodes a Mg^2+^ transporter. The position of the regulatory sequences that drive *Mma mgtC* expression is not known.

To investigate the role of *mgtC* in *M. marinum* pathogenesis, we generated a loss-of-function mutation by replacing the *mgtC* gene with a Hygro^r^ cassette *via* homologous recombination. The mutation was confirmed by PCR and Southern blotting (S1 Fig.). This mutation was complemented by introducing in the chromosome the wild-type *mgtC* gene as well as upstream sequence at the bacterial *att* site.

### Regulation of *Mma mgtC* expression by Mg^2+^ and growth of *mgtC* mutant in Mg^2+^ deprived medium

**Figure 2:**
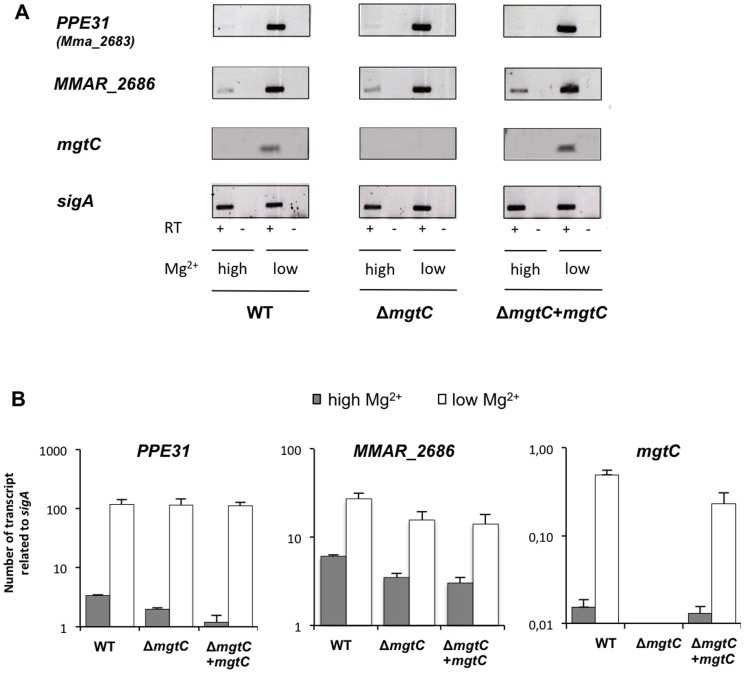
Expression of *Mma mgtC* and upstream genes in high Mg^2+^ and low Mg^2+^ conditions. (A) RT-PCR experiment on RNA isolated from *M. marinum* strains grown in high or low Mg^2+^ with primers specific for *mgtC*, *MMAR_2686*, *MMAR_2683* (PPE31) and *sigA*. Experiment was carried out with wild-type strain, *mgtC* mutant strain and complemented strain. Controls where reverse transcriptase was omitted (indicated by RT -) are done to verify the absence of genomic DNA contamination in the RNA sample. The *sigA* gene is used as control. (B) Quantification of *mgtC*, *MMAR_2686* and *MMAR_2683* RNA by Q-RT-PCR experiment using RNA isolated from *M. marinum* strains grown in high or low Mg^2+^. The sigma factor *sigA* was used as an internal standard. Results are expressed as means+standard deviations (SD) from a representative experiment performed in triplicate.

MgtC is highly induced by low Mg^2+^ concentrations in *S.* Typhimurium [11]. In *M. tuberculosis*, Mg^2+^ deprivation only slightly induced the *mgtC* gene (1.5 fold) whereas genes upstream of *mgtC* (Rv1806 through Rv1809) are clearly induced [13], [14]. *M. marinum* M strain was grown in Sauton's medium with or without Mg^2+^ and RNA was extracted to monitor the expression of *mgtC* along with two upstream genes: *MMAR_2686* that is located immediately upstream *mgtC* and *MMAR_2683* (*PPE31*), which is the first of the *PPE* genes. RT-PCR experiments indicated that expression of all three genes is highly induced by Mg^2+^ deprivation (Fig. 2A) whereas the control gene *sigA* is similarly transcribed in both conditions. Quantitative RT-PCR using *sigA* gene as internal control indicated an induction level by low Mg^2+^ of about 30 fold for *PPE31* and *mgtC* (Fig. 2B). The induction rate of MMAR_2686 is lower (about 5 fold), due to higher endogenous expression in high Mg^2+^ medium.

RNA extraction was also performed from *mgtC* mutant and complemented strain, to test the expression of *mgtC* from an ectopic location. As anticipated, the *mgtC* gene is not expressed in the *mgtC* mutant (whereas *PPE31* and *MMAR_2686* are expressed and regulated similarly than in the wild-type context) (Fig. 2). The *mgtC* gene is expressed and regulated by Mg^2+^ in the complemented strain to a level similar to the one found in the wild-type strain. This result demonstrates that *mgtC* is properly expressed and regulated at the *attB* locus in the complemented strain. Thus, upstream sequences present in the complementation vector (i.e included in the 840 bp upstream *mgtC*) are sufficient for Mg^2+^ regulation of *mgtC*.

**Figure 3:**
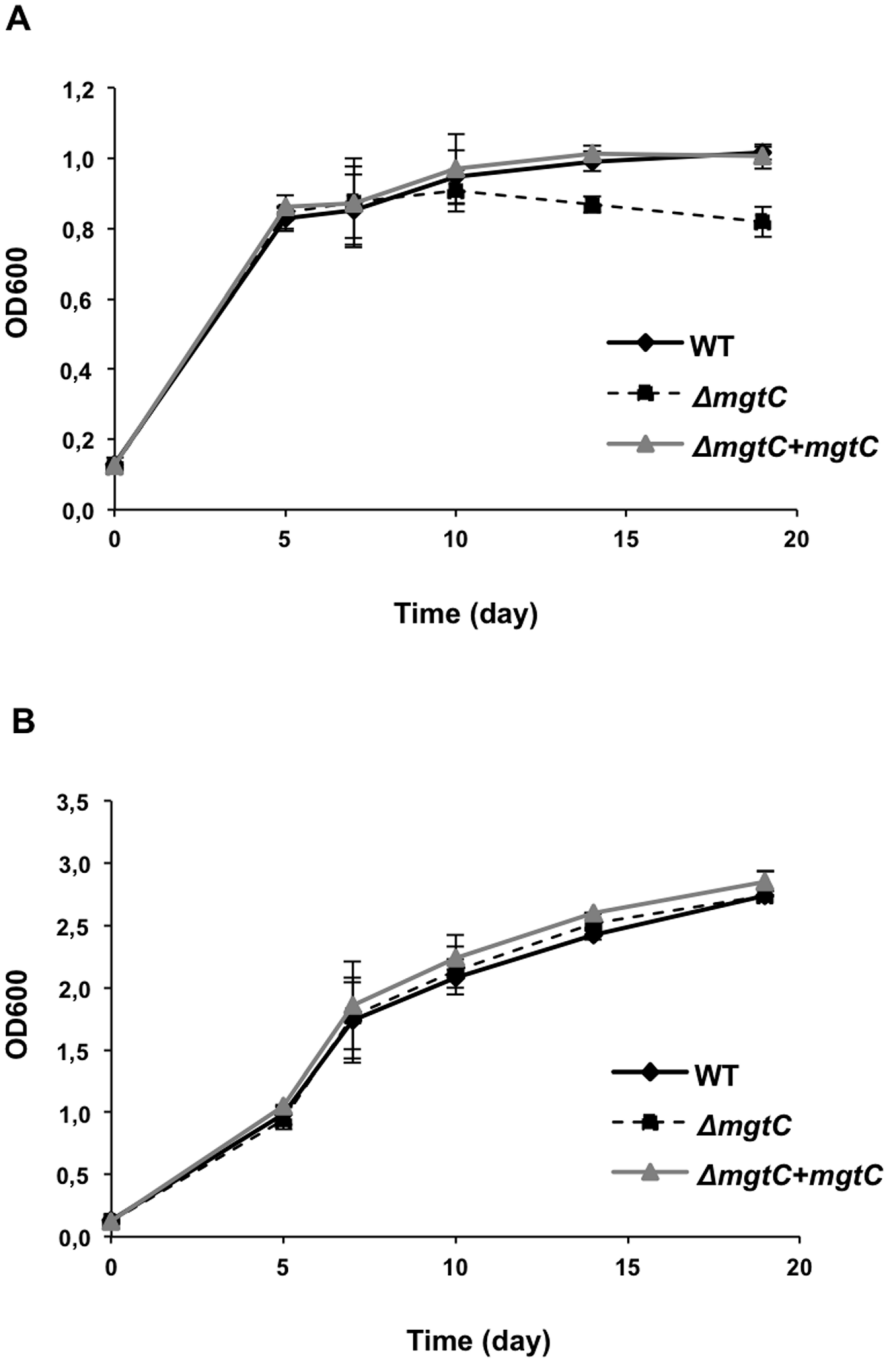
Growth of *Mma mgtC* mutant in Mg^2+^ deprived liquid medium. (A) Growth curves of *M. marinum* wild-type, Δ*mgtC* and Δ*mgtC*+*mgtC*::*attB* strains grown in Sauton's medium without magnesium. (B) or in regular Sauton's medium supplemented with magnesium. OD_600_ is indicated over the growth period. The curves from two independent experiments are shown with SD.

The growth rate of the *mgtC* mutant was evaluated in liquid cultures. The mutant shows a slight growth defect at late exponential phase in Mg^2+^-deprived broth medium (Fig. 3A), but not in medium supplemented with Mg^2+^ (Fig. 3B). As expected, the complemented strain behaves similarly to the wild-type strain in Mg^2+^-deprived medium, confirming the proper expression of the *mgtC* gene at the *attB* locus.

Together, these data indicate that MgtC is induced by Mg^2+^ deprivation and required for optimal growth in Mg^2+^-deprived medium in *M. marinum*. The results allowed validating the complementation of *mgtC* mutant by an extrachromosomal copy of the gene with its upstream DNA sequence.

### Behaviour of *mgtC* mutant upon intravenous infection in zebrafish embryos

**Figure 4:**
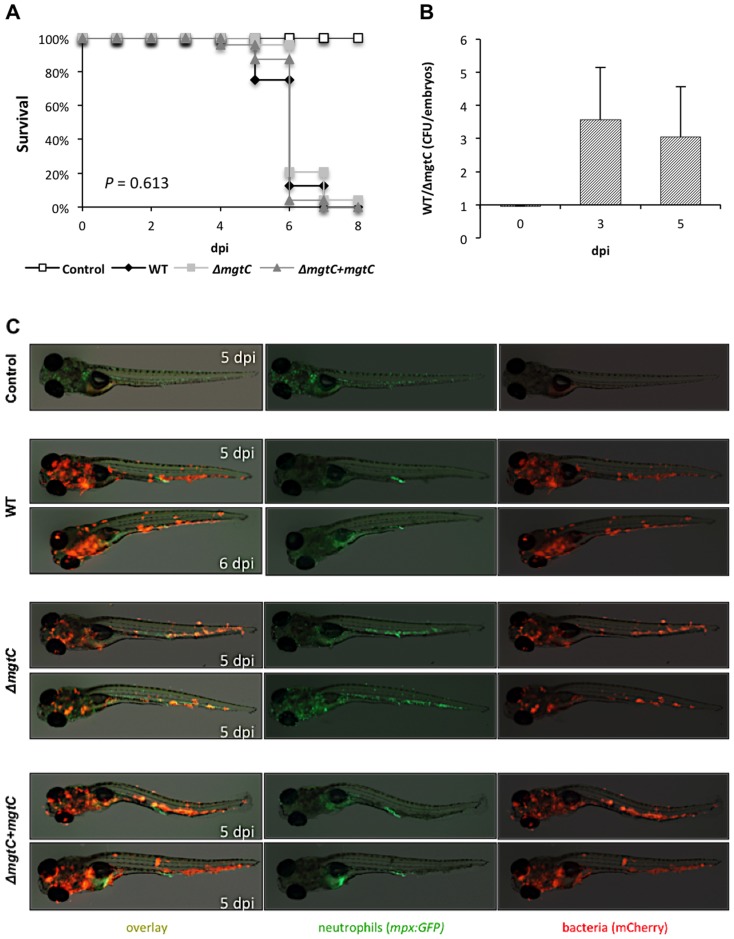
Intravenous infection of zebrafish with the *Mma mgtC* mutant. (A) Survival of 30 hpf embryos intravenously infected with 150–200 CFU of wild-type *M. marinum*, Δ*mgtC* mutant or complemented strain compared to non-injected controls (n = 24). Results are from a representative experiment (infection with 133 CFU for wild-type, 142 CFU for *mgtC* mutant and 205 CFU for complemented strain) out of three independent experiments. (B) Ratio of whole embryo bacterial counts between *Mma* M and *mgtC* mutant strain-infected embryos at 0, 3 and 5 dpi. A ratio of 1 indicates equal CFU values. A ratio >1 indicates that WT CFU are higher than *mgtC* mutant CFU. Results are expressed as mean CFU per embryo+SD from four independent experiments (0 and 5 dpi) or two independent experiments (3 dpi). The mild difference between mutant and wild-type strains is not statistically significant (Student Test). (C) Visualization of neutrophils in *mpx*:GFP infected larvae at late stages of infection (one day before embryo's death). Neutrophils fluoresce in green while *mcherry*-expressing bacteria fluoresce in red. Neutropenia occurs in wild-type and complemented strains but not in the *mgtC* mutant.

Studies were undertaken using the zebrafish infection model to probe the pathogenicity of the *Mma mgtC* mutant. *Mma*M, Δ*mgtC* mutant and Δ*mgtC*+*attB*::*mgtC* strains were transformed with pMV261_*mCherry* (S1 Table) and red fluorescent mycobacteria were injected intravenously (*iv*) in the Caudal Haematopoietic Tissue (CHT) in 30 hpf embryos. In this biological system, *iv*-injected mycobacteria are rapidly phagocytosed by circulating macrophages [29]. The infected embryos were monitored for survival and bacterial loads at different time points. The survival curves indicated that the virulence of the *mgtC* mutant is not significantly different from the parental *Mma* M or the complemented strains (Fig. 4A). The bacterial loads after 3 dpi or 5 dpi were slightly lower with the *mgtC* mutant, since less CFU were counted in embryos injected with the mutant strain comparatively to the wild-type strain (Fig. 4B). The number of neutrophils has been shown to dramatically decrease in zebrafish larvae unable to control bacterial proliferation upon injection with *Staphylococcus* or *Shigella* and neutropenia has been proposed to correlate with bacterial overgrowth [30], [31]. We took advantage of the *mpx*:GFP transgenic line (harbouring green fluorescent neutrophils) to follow the behaviour of neutrophils at late time of infection. By infecting *mpx*:GFP embryos with *M. marinum* strains, we observed the day before embryo's death that the increased number of bacteria is associated with a drastic decrease of green fluorescence in wild-type and complemented strains, indicative of a neutropenia (Fig. 4C). Interestingly, neutropenia was not observed with the *mgtC* mutant strain the day before embryo's death.

Overall, these results suggest that the *mgtC* mutant may not replicate as efficiently as the wild-type strain in zebrafish embryos, but that this effect is not sufficient to influence the outcome of the infection since embryos died similarly with both strains.

### The *Mma mgtC* mutant is more efficiently phagocytosed than its parental strain

**Figure 5:**
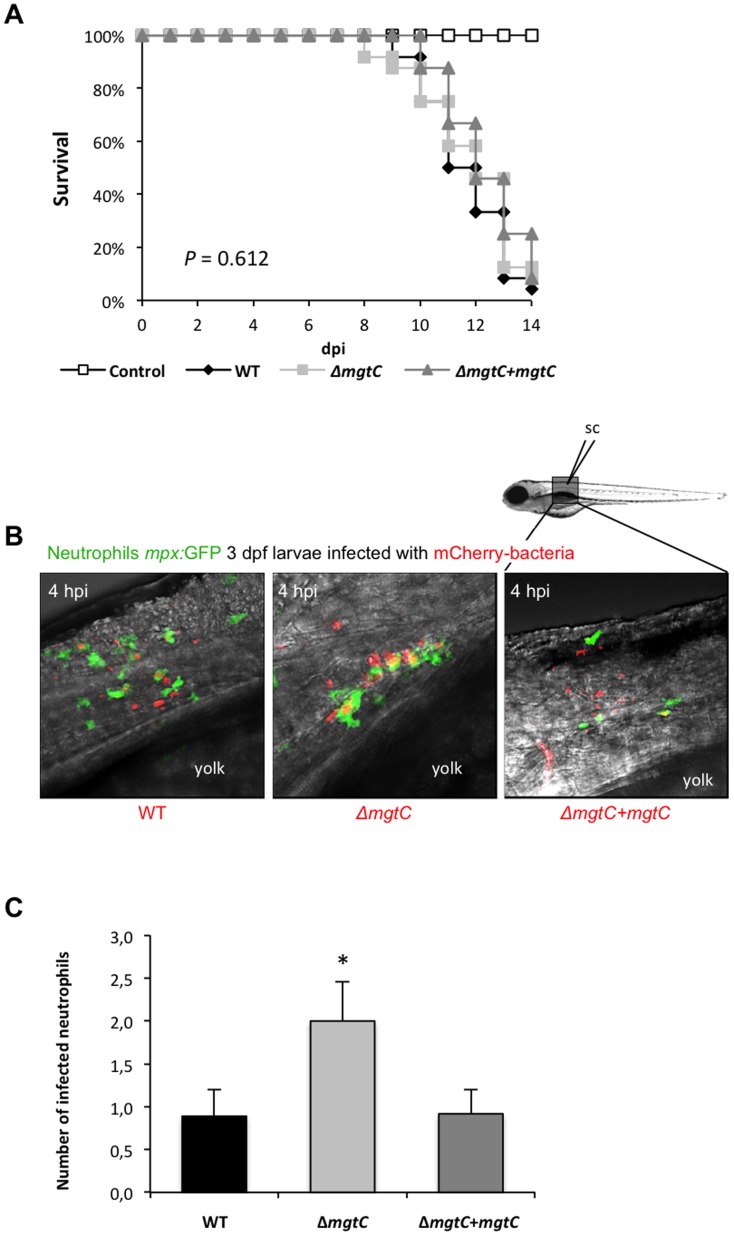
Increased phagocytosis of *mgtC* mutant by neutrophils upon subcutaneous injection of zebrafish embryos. (A) Survival of embryos subcutaneously injected at 3 dpf with approximately 50–100 CFU of wild-type *M. marinum*, *mgtC* mutant, complemented strain or PBS as control (n = 24). Results are from a representative experiment (infection with 119 CFU for wild-type, 74 CFU for *mgtC* mutant and 32 CFU for complemented strain) out of three independent experiments. A drawing of the injection site is shown. (B) Maximum intensity projection of neutrophil-phagocytosed bacteria at the site of injection, at 4 hpi by confocal microscopy. (C) Quantification of bacterial phagocytosis by neutrophils. The number of neutrophils containing phagocytosed bacteria at the site of injection was counted at 4 hpi using confocal microscope of a minimum of 10 embryos. Results are expressed+SD from a representative (104 CFU for wild-type, 82 CFU for *mgtC* mutant and 34 CFU for complemented strain) of three independent experiments. Asterisk indicates statistical significance (* *P*<0.05).

To further explore the behaviour of *Mma* strains towards neutrophils at early infection time, we carried out subcutaneous injections because it has been reported that, with this injection route, bacteria are directly taken up by neutrophils recruited at the infection site [32]. These previous subcutaneous experiments were performed using non-pathogenic *E. coli* and we report here for the first time subcutaneous injections of *Mma*. Comparing the death curves of embryos failed to show differences between the wild-type and mutant strains (Fig. 5A). Confocal microscopy was then used to study the recruitment of neutrophils at the early stage of infection (4 hpi). In agreement with the previous report on *E. coli* [32], we show here that neutrophils are recruited at the injection site and that mycobacteria are taken up by neutrophils upon sub-cutaneous injection (Fig. 5B). Interestingly, a higher proportion of red fluorescent bacteria within the green fluorescent neutrophils was detected with the mutant than with its parental or complemented strains, suggesting that the frequency at which the mutant is phagocytosed by neutrophils is higher than the other strains (Fig. 5B). The quantification of infected neutrophils confirmed a significant higher number for *mgtC* mutant, which was approximately twice that of the two reference strains (Fig. 5C).

**Figure 6:**
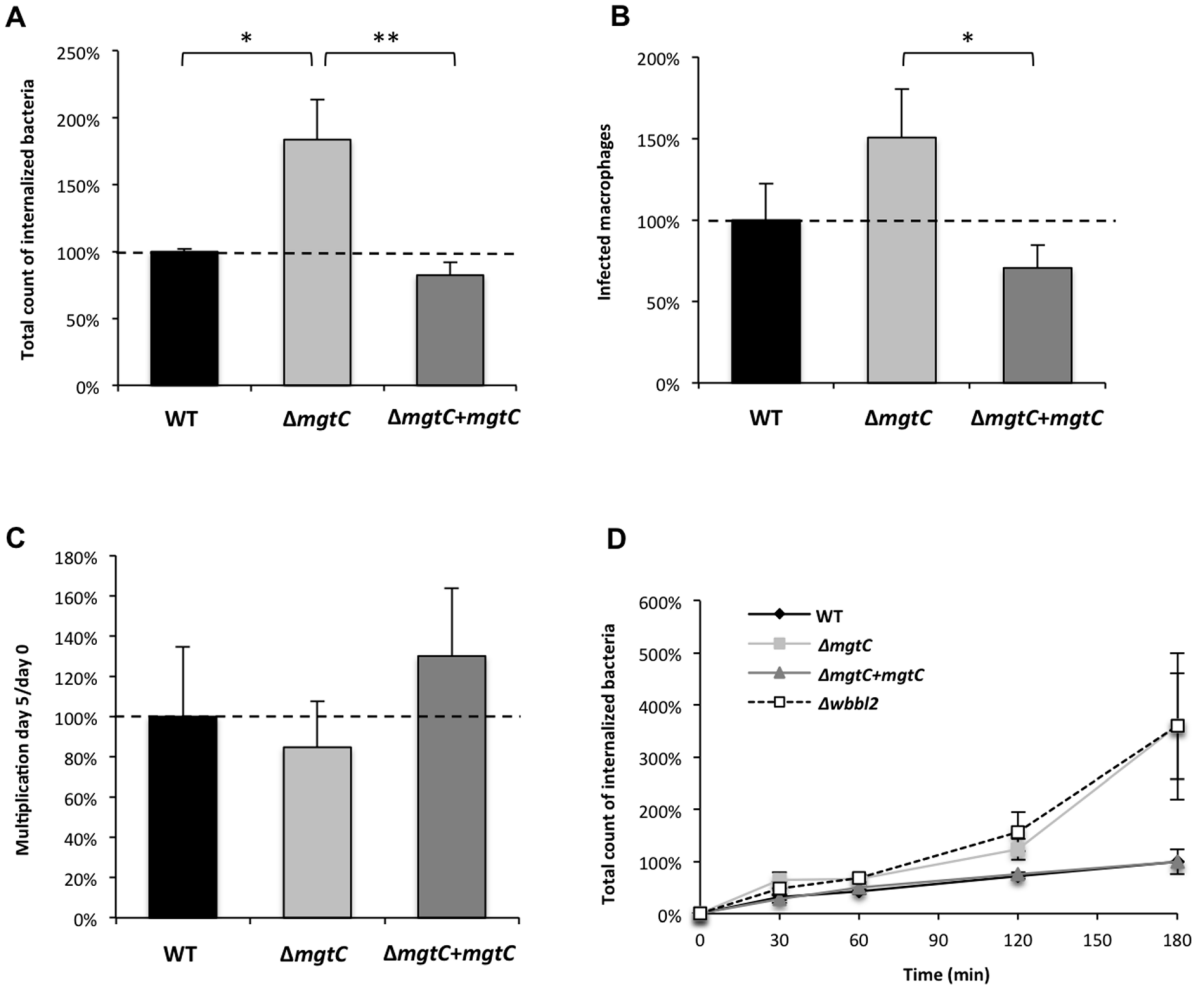
Phagocytosis and replication of the *mgtC* mutant in the J774 macrophage cell line. (A) Phagocytosis of the *mgtC* mutant and complemented strains by J774 macrophages is normalized to 100% for the wild-type strain. Results are expressed as means+SD from four independent experiments. (B) Numeration of macrophages infected with *mcherry*-expressing bacteria, normalized to 100% for the wild-type strain. Results are expressed as means+SD from three independent experiments. (C) Replication of the *mgtC* mutant and complemented strains by J774 macrophages normalized to the wild-type strain. Results are expressed as means+SD from four independent experiments. (D) Kinetic of phagocytosis of the *mgtC* mutant and complemented strains by J774 macrophages normalized to 100% for the wild-type strain at 180 min. The *wbbl2* mutant strain is used as a positive control. Results are expressed as means ± SD (error bars) from three independent experiments. Asterisks indicate statistical significance (* *P*<0.05; ** *P*<0.01).

**Figure 7:**
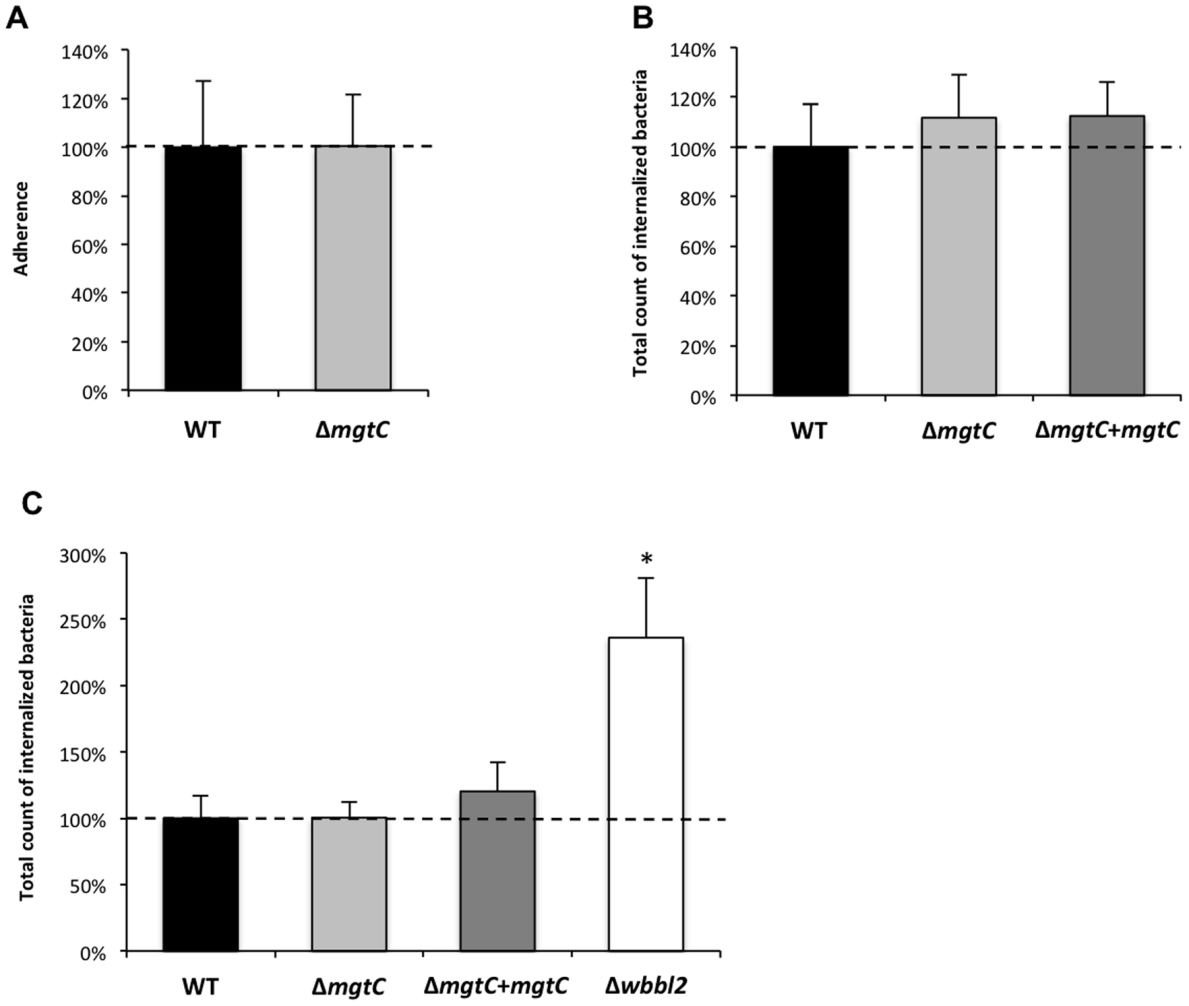
Adherence of the *mgtC* mutant on J774 macrophages and internalization in HeLa cells. (A) Adherence of the *mgtC* mutant strain to J774 macrophages after a 3-hr period of infection at 4°C, as compared to the WT strain. Results are normalized to 100% for the wild-type strain and expressed as means+SD from three independent experiments. (B) J774 macrophage internalization of the WT, the *mgtC* mutant and the complemented strains after a 3-hr period of infection in the presence of cytochalasin D (10 µg/ml). Results are normalized to 100% for the wild-type strain and expressed as means+SD from three independent experiments. (C) Internalization of the *mgtC* mutant and complemented strains in HeLa cells. The *wbbl2* mutant strain is included as a positive control. Results are normalized to 100% for the wild-type strain and expressed as means+SD from four independent experiments. Asterisks indicate statistical significance (* *P*<0.05).

To further investigate the behaviour of the *mgtC* mutant towards phagocytosis, experiments were carried out using phagocytic and non-phagocytic cells. Measurement of entry of bacteria into murine J774 macrophages indicated a two-fold increased uptake with the mutant strain as compared to the wild-type or complemented strains (Fig. 6A). The phagocytosis rate was next addressed by visualization of fluorescent bacteria and numeration of infected macrophages, leading to a similar pattern (Fig. 6B). An increased phagocytosis of similar magnitude was also observed upon infection of primary bone-marrow derived macrophages isolated from mice (BMDM) (data not shown). When the cells were lysed five days after infection to monitor the replication rate, the replication of the mutant appeared slightly lower than the other strains but the difference was not significant (Fig. 6C), which was also confirmed in BMDM (data not shown). We next analyzed the kinetic of *mgtC* mutant phagocytosis by including a *Mma* mutant defective in lipooligosaccharide (LOS) production (Δ*wbbl2* strain), which had been shown to be more efficiently phagocytosed by macrophages [26]. Both the *mgtC* and the *wbbL2* mutant share a highly similar kinetic profile (Fig. 6D). Differences with the parental strain appear more pronounced after 1 hr of infection, suggesting that inactivation of *mgtC* does not alter very early step of bacterial phagocytosis. Accordingly, when the experiment was carried out at 4°C to prevent active phagocytosis, no difference between the *mgtC* mutant and the parental strain was observed (Fig. 7A). A similar pattern was also observed following addition of cytochalasin D that prevents actin-driven phagocytosis (Fig. 7B). Hence, the increased phagocytosis of the mutant strain appears mediated by an actin-dependent process. Collectively, these results suggest that the higher phagocytic rate of *mgtC* mutant is not due to increased bacterial adherence to macrophages but is very likely due to a higher uptake of bacteria.

*Mycobacterium* species have been shown to have the ability to invade non-phagocytic cells, as epithelial cells [33], [34]. To investigate the behaviour of the *mgtC* mutant towards non-phagocytic cells, internalization experiments were performed using epithelial HeLa cells (Fig. 7C). Whereas the *wbbL2* mutant deficient for LOS synthesis shows higher internalization in HeLa cells, the *mgtC* mutant, as well as its parental and complemented counterpart, were equally internalized. From these results, it can be inferred that the phenotype of the *mgtC* mutant is restricted to professional phagocytes.

### Cell surface analysis

Whereas the function of MgtC in mycobacteria remains unknown, its recent identification as a protein that modulates ATP-synthase in *Salmonella* implies that MgtC may have pleiotropic effects. Previous studies in *Salmonella* indicated that the level of some outer membrane proteins are modulated in a *mgtC* mutant grown in low Mg^2+^ medium, which may be related with defect in bacterial division and cell elongation [35]. Bacterial surface plays a role in phagocytosis. In this respect, several mycobacterial (glycol)lipids are involved in many aspects of host pathogenesis [36], [37], including the internalization of bacteria by phagocytic and non-phagocytic cells. As mentioned above, LOS are bacterial surface molecules capable to modulate *Mma* phagocytosis [26], [38]. That the *wbbL2* and *mgtC* mutants are different toward internalization within epithelial cells suggests that *mgtC* mutant does not act by modulating expression of LOS. However, other cell wall-associated molecules, such as diacyltrehaloses (DAT) and polyacyltrehaloses (PAT), phtiocerol dimycocerosates (DIM) or phenolic glycolipids (PGL), have been reported to participate in *M. tuberculosis/M. leprae* phagocytosis [39]–[42].

To investigate the molecular basis that may explain the different phagocytosis rate of the *mgtC* mutant, we compared the profile of TDM/PGL, DIM and PAT in wild-type, *mgtC* mutant and complemented strain grown in Sauton's medium with or without Mg^2+^ (S2 Fig.). No significant differences were found between the three strains. If bacterial surface indeed differ between the wild-type and the mutant strains, this implies that differences rely on other surface molecules or that experimental conditions are not suitable to detect more subtle qualitative/quantitative differences.

## Discussion

MgtC appears as a unique virulence factor, shared by several intracellular bacterial pathogens, which, at least in *S.* Typhimurium inhibits bacterial's own F_1_F_o_ ATP synthase [10]. To further investigate the role of MgtC in mycobacteria, we have investigated its regulation and function in *Mma* virulence.

The transcription of the *Mma mgtC* gene and an upstream PPE gene (*PPE31*) is highly induced by Mg^2+^ limitation (about 30 fold). Complementation experiments demonstrated that the *mgtC* regulation is driven by a Mg^2+^-dependent regulatory element present between the end of PPE genes and the *mgtC* gene and do not rely on the *PPE31* upstream sequences. The regulation of *Mma mgtC* is similar to that of *S.* Typhimurium and *Y. pestis* but contrasts with that of *M. tuberculosis* where the *mgtC* gene is only slightly induced (1.5 fold) by Mg^2+^ deprivation [13], [14]. Hence, a strong regulation by Mg^2+^ is not restricted to cases where *mgtC* is co-transcribed with an Mg^2+^ transporter (as *S.* Typhimurium and *Y. pestis*). The fact that magnesium dependent expression of MgtC is conserved in phylogenetically distantly related bacteria is probably linked to the conserved function of MgtC in adaptation to magnesium fluctuations as indicated by the requirement of *Mma* MgtC for optimal growth in Mg^2+^-deprived broth medium. Despite the conserved role of *M. tuberculosis* MgtC for optimal growth in low Mg^2+^ media [5], the poor regulation of *Mtb mgtC* by Mg^2+^ suggests that *mgtC* regulation has evolved differently in this species. This could be related to the fact that *M. tuberculosis* is less exposed to environmental conditions, where magnesium concentrations are fluctuating, than non-tuberculous pathogens like *M. marinum* which have an external lifestyle and have to cope with various environmental changes.

MgtC is regarded as an intramacrophage multiplication factor in several intracellular bacterial pathogens that replicate in phagosomes, including *M. tuberculosis* [1]. However, we failed to detect a significant multiplication defect of the *Mma mgtC* mutant in either J774 macrophages or bone-marrow derived macrophages, even though a slight defect was observed. The lack of strong phenotype for the *Mma mgtC* mutant upon zebrafish embryos infection and intramacrophage replication may be related to the *Mma* intracellular niche. Even though *Mma* displays many similar virulence traits to *M. tuberculosis*, it exhibits also notable differences such as the ability to promote actin tail formation in the cytoplasm, probably to favor cell-to-cell spread [43]. *Mma* escapes from the phagosome rapidly and with a frequent rate [44], which may explain the lack of contribution of MgtC in intramacrophage replication. Another hypothesis to explain the discrepancy between the intracellular phenotypes of *M. tuberculosis* and *Mma mgtC* mutants may be related to the *M. tuberculosis* genetic background. Whereas a *mgtC* mutant constructed in the Erdman background exhibited an intramacrophage replication defect [5], an independent unpublished work reported in a review [45] failed to observe an intracellular growth defect for an *mgtC* mutant constructed in the H37Rv background, supporting the view that the genetic requirements and/or macrophage cell type may account for these differences.

The use of transgenic zebrafish embryos with fluorescent neutrophils allowed us to follow neutrophil behaviour *in vivo* upon *Mma* infection. Earlier studies demonstrated that neutrophils are very efficient to engulf *E. coli* on tissue surface but are virtually unable to phagocytose microbes in fluid environments [32]. Consistently, we confirm here that *Mma* can be phagocytosed by neutrophils shortly after infection upon subcutaneous injection, whereas neutrophils do not phagocytose *Mma* at initial site of infection when injected in the circulation [46]. After injection in the circulation, neutrophils are recruited to the granulomas where they phagocytize dying infected macrophages [46]. At later stages of infection, we observed a neutrophil depletion associated with bacteremia preceding the death of the larvae following infection with wild-type *Mma*. Neutropenia has also been reported in zebrafish embryos unable to control *Staphylococcus* or *Shigella* proliferation [30], [31]. This behaviour has been proposed to be a critical correlate of bacterial overgrowth [30], supported by the fact that in clinical infection, leukopenia is observed in overwhelming infections and is regarded as a poor prognostic sign [47]. Interestingly, neutropenia is not seen in embryos infected with the *Mma mgtC* mutant. The finding that bacterial loads in embryos are restricted with the mutant strain supports the idea of a direct link between neutropenia and the bacterial burden.

*Mma* MgtC is dispensable for intramacrophage replication, but we uncovered a novel role for MgtC in the early phase of macrophage infection. Our results indicate the *Mma mgtC* mutant is more efficiently phagocytosed than the wild-type strain by neutrophils upon subcutaneous infection of zebrafish embryos. This was subsequently confirmed in *ex vivo* experiments using various types of macrophages. In addition, this phenotype appears specific to phagocytic cells since no difference was found in epithelial cells, which contrasts with a LOS defective mutant that clearly showed increased uptake by macrophages and epithelial cells. In addition, kinetic experiments, as well as experiments carried out at 4°C, indicate that MgtC does not play a role in the initial attachment events of bacteria to macrophages but rather in later steps of the internalization process. Moreover, experiments carried out in the presence of cytochalasine D confirmed that this phenotype relies on an actin-based process. Cumulatively, our results demonstrate that the presence of MgtC limits the phagocytic process. Despite of its phagocytosis phenotype, the *mgtC* mutant is not attenuated in the zebrafish larvae infection model. This finding is consistent with other studies in *Mma* or *M. tuberculosis* mutants that also exhibited higher phagocytosis rate and were not correlated with an increased virulence phenotype in animal models [26], [39].

Several surface/cell wall components, including LOS, DAT/PAT, DIM and PGL, have been shown to modulate mycobacterial phagocytosis [36]. The glycan-rich outer layer of *M. tuberculosis* cell wall can act as an antiphagocytic capsule but its effect is mediated by limiting the association of the bacterium with macrophages [48], which thus differs from MgtC effect. Given the distinct phenotypes characterizing the *mgtC* and LOS mutants towards non-phagocytic cells, we propose that the differences reside unlikely in these glycolipids. DAT/PAT deficiency improved binding and entry of *M. tuberculosis* both in phagocytic and non-phagocytic cells, thus also differing from the phenotype of *Mma mgtC* mutant [39]. Interestingly, DIM deficiency reduced *M. tuberculosis* internalization in macrophages in an actin-dependent process, without affecting the bacterial binding to macrophages [40]. However, our TLC analysis failed to reveal major differences in DIM between the strains in the conditions tested. Moreover, no differences were found in the other lipids tested (DAT/PAT and PGL). Hence, experimental conditions may not be optimized to detect quantitative differences in those lipids or other surface molecules may be involved. Alternatively, the uptake phenotype may be driven by a mechanism that triggers signaling pathways of phagocytic receptors and/or early trafficking without noticeable bacterial surface modification.

In conclusion, our results indicate that the Mg^2+^ regulation of MgtC and its role for optimal growth in Mg^2+^-deprived media is conserved among bacteria that are not phylogenetically linked as *M. marinum* and *S.* Typhimurium. The role of MgtC in macrophages has been previously reported to be dissociated from its role in low Mg^2+^ medium [28]. This view is further substantiated by the present study, since *Mma* MgtC appears to have a role towards phagocytic cells linked to phagocytosis rather than intracellular multiplication. Even though the precise role of *Mma* MgtC during the infection process remains to be established, our results suggest that the involvement of MgtC towards professional phagocytes has evolved in bacterial pathogens, possibly to fit to the specific pathogen's lifestyles.

## Supporting Information

S1 Fig **Construction of** ***mgtC*** **mutant in** ***M. marinum*** **.** A) A DNA substrate for allelic replacement of the *M. marinum mgtC* gene was generated by cloning 979 bp upstream and downstream *mgtC* sequences to flank the *hyg^R^* gene. The locations of primers 1/1′, 2/2′ and 3/3′ used to check the *mgtC* mutant by PCR are indicated by arrows. Electrophoresis migration of PCR fragments 1 (primers 1/1′), 2 (primers 2/2′) and 3 (primers 3/3′) amplified from cultures of wild-type and *mgtC* mutant strain is shown. The upper lane indicates the 1569 bp DNA fragment cloned in the integrative vector pMV306 to complement the *Mma mgtC* mutant. B) Southern blot analysis of the *mgtC* mutant. The genomic structure of gene replacement mutant was examined by Southern blot analysis. Chromosomal DNA of wild-type and *mgtC* mutant strains were digested with *Xho*I and probed with either a segment of DNA of *mgtC* or the *hyg^R^* cassette. Hybridization signals at the expected size are detected (1792 bp for the *mgtC* probe in the wild-type strain and 3012 bp for *hyg* probe in the mutant strain).
(TIFF)

S2 Fig **Lipid profiles.** One-dimensional autoradiographic TLC of [1-^14^C]-propionate-labeled apolar lipids from *M. marinum* wild-type, *mgtC* mutant and complemented strains grown in Sauton's liquid medium A) or in Sauton's liquid medium without magnesium B). Equal amount (20,000 cpm) of radiolabeled lipids were spotted on TLC plates run in various solvents: chloroform/methanol (19∶1, v/v) for TDM/PGL, chloroform/methanol (99∶1, v/v) for PAT and petroleum ether/diethylether 9∶1 (v/v) for PDIM.
(TIFF)

S1 Table **Primers used in the study.**
(DOC)
