# Supplementary material for: Mycobacterium marinum MgtC Plays a Role in Phagocytosis but is Dispensable for Intracellular Multiplication
Source: PLoS One. 2014 Dec 29;9(12):e116052. doi: 10.1371/journal.pone.0116052 (PMC4278808; doi:10.1371/journal.pone.0116052)
Supplement: S1 Table — Primers used in the study. (DOC) [file pone.0116052.s003.doc]

| Primers | Sequences |
| --- | --- |
| pMV306-*Xba*I-5’ | GGTCTAGACAGCCAGGCTAGTGGGGA |
| pMV306-*Hind*III-3’ | CCCAAGCTTTCAGTCCGGCTCGGTGCC |
| Mma_ mgtC_qPCR_F | GGAGCCACGCTATTCGTCTT |
| Mma_ mgtC_qPCR_R | GCGCAAAGATCAAGTGTCCG |
| Mma_2686_qPCR_F | GGCTGTCTGCCATGATTTGG |
| Mma_2686_qPCR_R | ATCCGGGATTGTGGGTCTTC |
| PPE31_Mma_qPCR_F | CCGTAGAGGCCCAATACTGC |
| PPE31_Mma_qPCR_R | AGTGTTGGAAGACGCCAGTC |
| SigA_Mma_qPCR_F | ACGGTGATCGTGCGAAAAAC |
| SigA_Mma_qPCR_R | CGCGTAGGTGGAGAACTTGT |
| mgtCint-F | TGGTGGCCACCGGAGCCAC |
| mgtCint-R | CAACAGGATGTGGGCGGTC |
| pMV5’ | GCCCGGCCAGCGTAAGTAG |
| pJSC347-*Spe*I-up-5’ | GGACTAGTGTATCCATGCTGTGCACC |
| pJSC347-*Hind*III-up-3’ | CCCAAGCTTCGCTCGATGCCGATGAGG |
| pJSC347-*Xma*I-down-5’ | CCCCCCGGGGCTGGTGGCCGAGCTGTC |
| pJSC347-*Kpn*I-down-3’ | GGGGTACCGTCAGGTGGCCAAGCCCG |
